# The Unusual Aggregation and Fusion Activity of the Antimicrobial Peptide W-BP100 in Anionic Vesicles

**DOI:** 10.3390/membranes13020138

**Published:** 2023-01-21

**Authors:** Ana Rita Ferreira, Mariana Ferreira, Cláudia Nunes, Salette Reis, Cátia Teixeira, Paula Gomes, Paula Gameiro

**Affiliations:** 1 LAQV/REQUIMTE (Laboratório Associado para a Química Verde—Rede de Química e Tecnologia), Departamento de Química e Bioquímica, Faculdade de Ciências, Universidade do Porto, Rua do Campo Alegre, s/n, 4169-007 Porto, Portugal; 2 LAQV/REQUIMTE, Laboratório de Química Aplicada, Faculdade de Farmácia da Universidade do Porto, Portugal, Rua de Jorge Viterbo Ferreira, 228, 4050-313 Porto, Portugal

**Keywords:** cationic antimicrobial peptides, bacterial membranes, large unilamellar vesicles, giant unilamellar vesicles, Förster resonance energy transfer, membrane fusion, confocal microscopy

## Abstract

Cationic antimicrobial peptides (CAMPs) offer a promising strategy to counteract bacterial resistance, mostly due to their membrane-targeting activity. W-BP100 is a potent broad-spectrum cecropin-melittin CAMP bearing a single N-terminal Trp, which was previously found to improve its antibacterial activity. W-BP100 has high affinity toward anionic membranes, inducing membrane saturation at low peptide-to-lipid (P/L) ratios and membrane permeabilization, with the unique property of promoting the aggregation of anionic vesicles only at specific P/L ratios. Herein, we aimed to investigate this unusual behavior of W-BP100 by studying its aggregation and fusion properties with negatively-charged large (LUVs) or giant (GUVs) unilamellar vesicles using biophysical tools. Circular dichroism (CD) showed that W-BP100 adopted an α-helical conformation in anionic LUVs, neutralizing its surface charge at the aggregation P/L ratio. Its fusion activity, assessed by Förster resonance energy transfer (FRET) using steady-state fluorescence spectroscopy, occurred mainly at the membrane saturation/aggregation P/L ratio. Confocal microscopy studies confirmed that W-BP100 displays aggregation and detergent-like effects at a critical P/L ratio, above which it induces the formation of new lipid aggregates. Our data suggest that W-BP100 promotes the aggregation and fusion of anionic vesicles at specific P/L ratios, being able to reshape the morphology of GUVs into new lipid structures.

## 1. Introduction

Antimicrobial resistance is one of the major threats of the 21st century, impacting the global health and economy [1,2]. The overuse and misuse of antibiotics in the last decades and the high plasticity of bacteria to circumvent the action of antimicrobial agents have triggered the urgent need for new agents, effective against multi-drug resistant (MDR) infections, especially those caused by the so-called ESKAPE (*Enterococcus faecium*, *Staphylococcus aureus*, *Klebsiella pneumoniae*, *Acinetobacter baumannii*, *Pseudomonas aeruginosa*, and *Enterobacter* spp.) group of priority pathogens [2,3].

Cationic antimicrobial peptides (CAMPs) are short molecules found in the immune system of several organisms such as humans, amphibians, plants, and bacteria, usually exhibiting a broad-spectrum of action and selectivity to bacteria [4,5]. Therefore, CAMPs have widely been explored as a promising alternative to conventional antibiotics. These molecules target and permeabilize the membrane of bacteria, being less susceptible to the development of bacterial resistance mechanisms [6]. In addition, their chemical properties promote electrostatic and/or hydrophobic interactions with the bacterial membranes (negatively charged), favoring their membrane penetration [4,6].

Cecropin A-melittin hybrid peptides are known to exhibit potent antibacterial and membrane targeting activities, being promising candidates to fight bacterial infections. W-BP100 (WKKLFKKILKYL-NH_2_) [7] is a cecropin A-melittin hybrid CAMP, resultant from the addition of a tryptophan (Trp) residue in the *N*-terminus of BP100 [8,9,10]. The addition of a single Trp residue to BP100 enhanced and broadened its antibacterial activity against Gram-negative and Gram-positive bacteria [7], as previously reported for other CAMPs containing Trp residues [11,12,13,14]. W-BP100 inhibited the growth of susceptible reference bacterial strains at low MIC values (0.75−3 μmol dm^−3^ against the Gram-negative strains of *Escherichia coli* and *Pseudomonas aeruginosa*; and 1.5−3.0 μmol dm^−3^ against the Gram-positive strains of *Staphylococcus aureus* and *Enterococcus faecalis*), improving by 36- to 72-fold the antibacterial activity of BP100 against Gram-positive bacteria. Additionally, the peptide was found to be bactericidal at the MIC against all of the tested bacterial strains.

W-BP100 also demonstrated a higher affinity for anionic large unilamellar vesicles (LUVs), characteristic of bacterial membranes, inducing membrane saturation and penetration into anionic and zwitterionic lipid vesicles [7]. Moreover, W-BP100 was proposed to induce the aggregation of liposomes at a peptide-to-lipid (P/L) ratio near membrane saturation conditions (P/L ratio of ~0.06), followed by disaggregation above that P/L ratio. This disaggregation phenomenon was not previously observed for its precursor (BP100) [7,8,11]. Instead, W-BP100 reduced the vesicle size at a P/L ratio close to the membrane saturation conditions (P/L ranging between 0.07 and 0.11), while no significant size alterations were detected at higher P/L ratios. W-BP100 was found to promote a strong and fast membrane permeabilization in anionic LUVs, suggesting a mechanism of action (MOA) initiated by (1) electrostatic-driven adsorption of W-BP100 to the surface of anionic LUVs, followed by the reduction in the membrane surface charge and a detergent-like action (close to the critical P/L ratio at which membrane saturation occurs); and possibly followed by (2) peptide translocation, translating into a sequential aggregation and disaggregation of the anionic LUVs [7]. Nevertheless, the effect and overall MOA of W-BP100 on anionic LUVs at higher P/L ratios remain unexplored.

Membrane fusion is part of various biological processes such as the entry of enveloped viruses into host cells, fertilization, cell division, or synaptic transmission, and it is attributed to the action of fusogenic peptide sequences, usually belonging to the structure of larger proteins [15]. Membrane-active peptides, particularly cell-penetrating peptides (CPPs) as well as some CAMPs, might also elicit the ability to induce membrane and vesicle fusion [16,17]. The commonly accepted MOA of membrane fusion, called the lipid stalk model, is initiated by a docking step that involves (i) the contact of two membranes; (ii) merging of two apposed lipid bilayers into a continuous one, called the stalk; and (iii) transmembrane fusion, followed by (iv) content mixing of the respective lipid vesicles through pore formation [15,16]. Membrane fusion can be monitored using Förster resonance energy transfer (FRET), a photophysical process where an electronically excited fluorophore molecule, the donor, transfers its excitation energy to an acceptor fluorophore in the electronic ground state. The occurrence of FRET implies that the absorption spectrum of the acceptor overlaps the emission spectrum of the donor and is dependent on the intermolecular distance between both molecules (given by the Föster radius) [18,19]. The study of lipid mixing and membrane fusion can be performed using NBD- and Rho-labelled phospholipids, a classic FRET pair of fluorophores [19].

In this work, we aimed to further explore the aggregation and fusion activities of W-BP100 in both anionic LUVs and giant unilamellar vesicles (GUVs) composed of 1-palmitoyl-2-oleoyl-*sn*-glycero-3-phosphocholine (POPC) and 1-palmitoyl-2-oleoyl-*sn*-glycero-3-phosphoglycerol (POPG) at a 1:1 molar ratio using biophysical tools. 

Circular dichroism (CD) spectroscopy analysis revealed that W-BP100 adopted an α-helical conformation in anionic POPC:POPG (1:1) LUVs independent of the P/L ratio, but such conformation was partially lost under vesicle aggregation conditions. Aggregation studies using dynamic light scattering (DLS) and nanoparticle tracking analysis (NTA) confirmed that W-BP100 induces the aggregation of anionic POPC:POPG (1:1) LUVs only at critical P/L ratios, while the same effect was not observed at high P/L, at which vesicles retained the same size of those not exposed to the peptide. Moreover, vesicle aggregation was explained by electroneutralization of the membrane surface charge, as confirmed by the zeta potential analysis. The aggregation and membrane fusion properties of W-BP100 were also explored in LUVs by steady-state fluorescence spectroscopy, using FRET, and in GUVs by confocal microscopy. FRET studies showed vesicle fusion mostly at the membrane saturation and aggregation conditions. Confocal microscopy experiments revealed the aggregation of anionic GUVs by the action of W-BP100 close to the membrane saturation P/L ratio, followed by vesicle rearrangement into amorphous lipid-like structures. These results not only corroborate previous vesicle aggregation studies [7], indicating that W-BP100 induces changes in the GUV morphology, reshaping their structure above a critical P/L ratio. This unusual behavior was not reported for its parental CAMP, BP100 [20,21], suggesting a different MOA for W-BP100.

## 2. Materials and Methods

### 2.1. Chemical Reagents

Fmoc-Rink Amide MBHA resin, *N*,*N*,*N*,*N’*-tetramethyl-O-(1H-benzotriazol-1-yl)uranium hexafluorophosphate (HBTU), and O-(1H-6-chlorobenzotriazole-1-yl)-1,1,3,3-tetramethyluronium hexafluorophosphate (HCTU) were purchased from Novabiochem, Merck, Darmstadt, Germany. Dimethylformamide (DMF), piperidine (HPLC grade), *N*-ethyl-*N*,*N*-diisopropylethylamine (DIEA), *N*-methylmorpholine (NMM), trifluoroacetic acid (TFA), triisopropylsilane (TIS), phenol, chloroform, 4-(2-hydroxyethyl) piperazine-1-ethanesulfonic acid hemisodium salt (HEPES), sodium chloride (NaCl), sodium fluoride (NaF), dipotassium hydrogen phosphate, sodium hydrogen phosphate (Na_2_HPO_4_), sodium dihydrogen phosphate (NaH_2_PO_4_), ascorbic acid, and sucrose were acquired from Sigma-Aldrich (Saint Louis, MO, USA). D-(+)-glucose anhydrous from Biochemopharma (Cosne-Cours-sur-Loire, França), dichloromethane (DCM) from Fischer Scientific (Hampton, NH, USA), *N*-Fmoc-protected amino acids (Fmoc-AA-OH) from Bachem (Bubendorf, Switzerland), *tert*-butyl methyl ether (MTBE) and hexa-ammonium heptamolybdate-4-hydrate from Honeywell Riedel-de Haën^TM^ (Seelze, Germany), and acetonitrile (ACN) from CARLO ERBA Reagents, Barcelona, Spain were also used. Lipids 1-palmitoyl-2-oleoyl-glycero-3-phosphocholine (POPC), 1-palmitoyl-2-oleoyl-*sn*-glycero-3-phospho-(10-*rac*-glycerol) (POPG), 1-palmitoyl-2-oleoyl-*sn*-glycero-3-phosphoethanolamine (POPE), 1,2-dioleoyl-*sn*-glycero-3-phosphoethanolamine-*N*-(7-nitro-2-1,3-benzoxadiazol-4-yl) (ammonium salt) (NBD-DOPE), 1,2-dipalmitoyl-*sn*-glycero-3-phosphoethanolamine-*N*-(7-nitro-2-1,3-benzoxadiazol-4-yl) (ammonium salt) (NBD-DPPE), and 1,2-dipalmitoyl-*sn*-glycero-3-phosphoethanolamine-*N*-(lissamine rhodamine B sulfonyl) (ammonium salt) (Rho-DPPE) were obtained from AVANTI Polar Lipids Inc. (Alabaster, AL, USA).

Biophysical studies were performed in HEPES buffer (10 mmol dm^−3^, 150 mmol dm^−3^ NaCl, in milli-Q water; pH 7.4), to mimic the physiological ionic strength [7,11]. For the CD studies, all solutions were prepared in sodium phosphate buffer (9 mmol dm^−3^ Na_2_HPO_4_, 2 mmol dm^−3^ NaH_2_PO_4_, 150 mmol dm^−3^ NaF, in milli-Q water; pH 7.4).

### 2.2. Synthesis of W-BP100

The peptide W-BP100 (WKKLFKKILKYL-NH_2_) was manually synthesized by solid-phase peptide synthesis (SPPS) using the standard 9-fluorenylmethoxycarbonyl (Fmoc)/*^t^*Bu orthogonal protection scheme, on a Fmoc-Rink Amide MBHA resin (100–200 mesh, 0.52 mmol g^−1^), as previously described by Ferreira et al. [7].

The compounds were purified through preparative reversed-phase high-performance liquid chromatography (RP-HPLC) on a Hitachi-Merck LaPrep Sigma system (VWR, Radnor, PA, USA) equipped with an LP3104 UV detector and an LP1200 pump, employing a reverse-phase C18 column (250 × 25 mm ID and 5 µm pore size, Merck, Kenilworth, NJ, USA), and the purity was verified in an analytical HPLC from Hitachi–Merck LaChrom Elite (VWR) system equipped with a quaternary pump, a thermostatted automated sampler, and a diode array detector. Analyses were performed with a reverse-phase C18 column (150 × 4.6 mm ID and 5 µm pore size, Merck) at a 1 mL min^−1^ flow rate.

The molecular weight of W-BP100 was confirmed in deionized water using electrospray ionization-ion trap mass spectrometry (ESI-IT MS). The compounds were analyzed in a LTQ Orbitran XL from Thermo Scientific Fischer, Bremen, Germany, controlled by a LTQ Tune Plus 2.5.5 and using the Xcalibur 2.1.0 software.

The peptide was quantified by UV–Vis spectroscopy using the Thermo ScientificTM NanoDrop^TM^ One/OneC Microvolume UV–Vis spectrophotometer (Thermo Fisher Scientific, Waltham, MA, UAS).

### 2.3. Liposome Preparation

#### 2.3.1. Preparation of LUVs

LUVs were used as the mimetic model membrane systems of bacteria. LUVs of POPC:POPG (1:1), POPE:POPG (1:1), and POPC:POPG (3:1) were prepared as previously described by Ferreira et al. [7] by thin-film hydration followed by the extrusion method [22]. Briefly, adequate amounts of phospholipids were dissolved in chloroform and dried under a nitrogen stream. The obtained lipid films were evaporated under vacuum, and protected from light for 3 h to ensure the total remove of organic solvent. Multilamellar vesicles (MLVs) were obtained by the redispersion of the lipid films in HEPES buffer. The MLVs were exposed to five freeze–thaw cycles and finally equilibrated at 25.0 ± 0.1 °C for 30 min—above the phase transition temperature of the lipids (−2 °C for POPC and POPG and 25 °C for POPE [23]). LUVs were obtained by 10 times extrusion of the MLV suspensions through polycarbonate filters with a 0.1 µm pore size (Whatman, GE Healthcare, Maidstone, UK) on a Lipex biomembrane extruder (LIPEX Biomembranes, Vancouver, BC, Canada) attached to a CLIFTON thermostatic circulating water bath. The size distribution of the LUVs was characterized by DLS at 37.0 ± 0.1 °C (Zetasizer nanoZS, Malvern Instruments, Malvern, UK), using a He-Ne laser (λ = 633 nm), a fixed scattering angle of 173°, refractive indices of 1.330 for HEPES (water) and 1.400 for lipids, and viscosity of the dispersant of 0.6913 cP (water).

The average particle size of the LUVs was ~100 nm, with a polydispersity index (PdI) <0.1. LUV suspensions were stored at 4 °C and protected from light prior to use.

The phospholipid concentration in the liposome suspensions was estimated by phosphate analysis through a modified Bartlett method [24,25].

#### 2.3.2. Preparation of GUVs

GUVs made of POPC:POPG (1:1) lipids were prepared by the electroformation method [26,27]. Fluorescently labelled lipids (FLLs) were used to stain the membrane of GUVs to perform the vesicle fusion assays. Lipid stocks (10 mmol dm^−3^) of POPC:POPG (1:1) containing FLLs (2% (mol/mol) of NBD-DPPE or 0.1% (mol/mol) of Rho-DPPE) were dissolved in chloroform:methanol (9:1 *v*/*v*) and stored protected from light at −20 °C for further use.

GUV formation was performed in a Vesicle Prep Pro device (Nanion Technologies, München, Germany) by automated preparation using indium tin oxide (ITO)-coated surface glasses. The surface conductivity of the ITO-coated glass slides was verified (<100 Ω, the optimal value to proceed with the GUV formation protocol). A thin lipid film was formed on the conductive side of an ITO-coated glass by slowly spreading 20 µL of lipid solution (stock solution of 10 mmol dm^−3^) on the slide surface, and the lipid film was air-dried for 1 min to allow for the evaporation of organic solvents. The lipid film was surrounded by an 18-mm diameter O-ring fixed to the glass surface with grease, both supplied by the manufacturer. The lipid film was then hydrated with 280 µL of 280 mmol dm^−3^ sucrose aqueous solution, and a second ITO-coated glass was placed on top with the conductive side facing downward of the lipid film, ensuring no air bubble formation. Upon sealing the Vesicle Prep Pro device, the GUV formation was performed at 5 Hz for a total of 135 min by initially increasing the voltage from 0 to 3 V for 5 min, allowing for an electroformation step with an alternating voltage of 3 V for 120 min, and finally, a decrease in voltage from 3 to 0 V to promote GUV detachment. The freshly prepared GUVs were placed in a centrifuge tube, diluted with 560 µL of the same sucrose solution (~3 times dilution), stored at 4 °C, and used within 1 week. The POPC:POPG (1:1) GUVs were obtained with an average size diameter ranging from 3 to 6 μm.

### 2.4. Secondary Structure of W-BP100

The CD spectroscopy measurements were carried out in a Jasco J-815 spectropolarimeter equipped with a PFD 425S Jasco Peltier temperature controller system (Japan Spectroscopy Co., Tokyo, Japan), under a nitrogen gas flow of 8 L h^−1^ at 20.00 ± 0.01 °C, using a quartz cuvette with a 1-mm optical length. The CD spectra were recorded in the wavelength range of 190–260 nm, bandwidth of 1 nm, scanning speed of 50 nm/min, data integration time of 2 s, and 16 accumulations. Data collection and data analysis were performed using Spectra Manager 2 software.

The secondary structure of W-BP100 was assessed by CD in a sodium phosphate aqueous buffer and in the presence of POPC:POPG (1:1) LUVs. A total of 40 µmol dm^−3^ (0.064 mg mL^−1^) of W-BP100 was mixed with 100 µmol dm^−3^ LUVs at different P/L ratios (0, 0.03, 0.06, 0.09, 0.12, and 0.15) and incubated for 10 min at room temperature before sample measurement. The buffer (blank) was subtracted to the CD spectra of all samples. CD data, given in units of ellipticity (θ), were measured in millidegrees (mdeg) and converted to molar ellipticity per residue $\left[\theta \right]\;\mathrm{by}$ using the following equation [28]:

(1) $$\left[\theta \right]=\frac{\theta }{10\times c\times n\times l}$$

where $\left[\theta \right]$ is expressed in deg cm^2^ dmol^−1^; c is the molar concentration; n is the number of residues; and l is the cuvette optical length in cm. Two independent experiments were performed.

The mean helix content or helicity ($f_{H}$) was calculated according to Luo and Baldwin using Equation (2) [29]:

(2) $$f_{H}=\frac{{\left[\theta \right]}_{222}-\theta_{C}}{\theta_{H}-\theta_{C}}$$

where [$\theta {]}_{222}$ is the molar ellipticity per residue at 222 nm; $\theta_{C}$ and $\theta_{H}$ are the baseline ellipticities for random coil and complete helix, respectively. These are given by the following formulas: $\theta_{C}=2220-53T$ and $\theta_{H}=(-44000+250T)/\left(1-3/N\right)$, where T is temperature in °C, and N is the peptide length in number of residues [30]. 

### 2.5. Zeta Potential Analysis of LUVs

The zeta potential of LUVs was determined by measuring the electrophoretic light scattering (ELS) in a Zetasizer Nano ZS (Malvern Instruments, Malvern, UK). The POPC:POPG (1:1) LUVs were exposed to 0, 6, 9, 12, 15, and 18 μmol dm^−3^ of W-BP100 in filtered HEPES buffer (using a syringe filter with 0.2 µm pore size) for 10 min. Samples were measured with light detection at 17° using the backscatter mode, in disposable folded capillary cells at 25.0 ± 0.1 °C [8]. Zeta potential was determined as the average of five measurements with 10–30 runs per measurement.

### 2.6. Aggregation and Fusion of LUVs

#### 2.6.1. Vesicle Aggregation Studies by DLS and NTA

The effect of W-BP100 on the size of the LUVs was assessed by DLS [7,8]. W-BP100 was mixed with 100 µmol dm^−3^ POPC:POPG (1:1) LUVs in filtered HEPES buffer at different P/L ratios (0, 0.03, 0.06, 0.09, 0.12, and 0.15) for 10 min at room temperature. The vesicle size distribution was followed over time (24 h and 1 week after incubation) to evaluate the stability of LUVs in the presence of the peptide. Samples were analyzed in 1-cm path length plastic cuvettes using a 173° backscatter angle, upon an equilibration time of 60 s. The refractive indices used were 1.330 (water) and 1.400 (lipids) and the viscosity of the dispersant used was 0.888 cP (water). The average particle size diameter and particle size distribution were measured at 25.0 ± 0.1 °C, and data were presented as the intensity-weighted and number-weighted size distribution.

The vesicle size distribution was similarly evaluated by NTA (Malvern Panalytical NanoSight NS300 instrument, equipped with a 642 nm laser module, Malvern, UK) following the protocol described by Bessa et al. [31]. Samples were diluted 1:50 in filtered HEPES buffer to ensure an optimal LUV concentration for vesicle detection (~2 µmol dm^−3^), and 1 mL of the sample was injected into the equipment’s flow cell. The focus and camera level were adjusted to obtain the best possible image following the guidelines supplied by the manufacturer. A total of ten videos of 60 s each were recorded at 25.0 ± 0.1 °C, considering a dispersant viscosity of 0.888 cP (water). Videos were captured at different image fields of the injected sample volume to ensure the detection of many particles. Data collection and analysis were performed using the NTA 3.2 software to obtain the hydrodynamic diameter distributions and the vesicle concentration in each sample. The videos were analyzed independently by adjusting the detection threshold settings according to the scattered light intensity observed in the captured videos.

#### 2.6.2. Vesicle Fusion Studies Using FRET

Vesicle fusion studies were performed using a FRET-based assay using two methods: the fluorophore mixing method and the fluorophore dilution method.

For the fluorophore mixing method, two sets of LUVs of POPC:POPG (1:1) were prepared with 0.9% molar fraction of FLLs, NBD-DOPE (donor), or Rho-DPPE (acceptor). The LUVs containing 0.9% NBD-DOPE were then mixed with 0.9% Rho-DPPE LUVs at a 1:1 molar ratio (A0). For the fluorophore dilution method, LUVs of POPC:POPG (1:1) containing a 0.9% molar fraction of NBD-DOPE and 0.9% molar fraction of Rho-DPPE were mixed with unlabelled LUVs at a 1:2 ratio (A0).

W-BP100 was then added at different P/L ratios (0, 0.03, 0.09, 0.12, 0.15, and 0.18) by using a total lipid concentration of 100 µmol dm^−3^ (A1–A6). Fluorescence emission spectra were recorded upon 10 min of incubation, under stirring, at 25.0 ± 0.1 °C, at an excitation wavelength of 460 nm (donor), in the wavelength range of 480–650 nm and using excitation/emission slits of 5/5 nm (fluorophore mixing method) and 5/6 nm (fluorophore dilution method). LUVs containing 0.9% NBD-DOPE were used to determine the fluorescence intensity of the donor in the absence of the acceptor for each method. Two independent experiments were performed for each experimental methodology and the efficiency of energy transfer or FRET efficiency (*E*) was determined according to Equation (3) [32]:

(3) $$E=1-\frac{I_{\mathrm{DA}}}{I_{D}}$$

where $I_{\mathrm{DA}}$ and $I_{D}$ are the fluorescence intensities in the presence and absence of the acceptor, respectively. 

### 2.7. Aggregation and Fusion of GUVs by Confocal Microscopy

For the confocal microscopy studies, POPC:POPG (1:1) GUVs were prepared with 2% NBD-DPPE or 0.1% Rho-DPPE GUVs, as described in Section 2.3.2. To evaluate the effect of W-BP100 on the membrane integrity and aggregation, 2% NBD-labelled GUVs were used, while for the vesicle fusion assays, 2% NBD-labelled GUVs were mixed with 0.1% Rho-labelled GUVs at a 1:1 molar ratio.

GUVs were observed under confocal laser scanning microscopy in a µ-Dish 35 mm Petri dish from Ibidi® and the images were recorded on a Leica Stellaris 8 confocal microscope (Leica Microsystems, Wetzlar, Germany) equipped with the Leica Application Suite X package (LAS X, Wetzlar, Germany). GUVs (~238 μmol dm^−3^) prepared in 280 mmol dm^−3^ sucrose were first diluted five times in a 280 mmol dm^−3^ glucose aqueous solution to allow for vesicle deposition on the bottom of the Petri dishes due to a density gradient. A total of 40 µL of GUVs (~48 µmol dm^−3^) was placed on Petri dishes and small aliquots of a peptide stock solution were successively added at a concentration range of 40–120 µmol dm^−3^ (corresponding to P/L of 0, 0.8, 1.2, 2.1, 2.5). Images were acquired under a 63×-oil objective, over 10 min, with 10 s intervals between frames. Image analysis and the size of the GUV at different P/L ratios were performed using ImageJ software (version 1.53f51, U. S. National Institutes of Health, Bethesda, MD, USA).

## 3. Results and Discussion

In the present work, we further explored the contribution of the single Trp of W-BP100 on the size, aggregation, stability, and fusion of anionic POPC:POPG (1:1) LUVs and GUVs using CD, zeta potential, DLS, NTA, fluorescence spectroscopy, and confocal microscopy.

### 3.1. Secondary Structure of W-BP100 in Anionic LUVs

**Figure 1:**
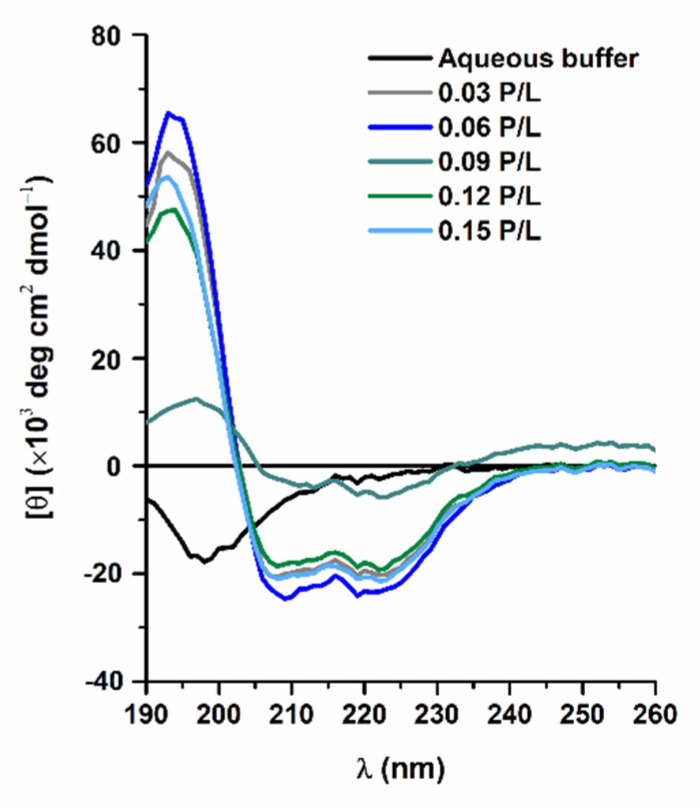
Far-UV CD spectra of W-BP100 in aqueous buffer and in the presence of anionic LUVs. Representative spectra of molar ellipticity per residue of W-BP100 in POPC:POPG (1:1) LUVs at increasing P/L ratios. Data were obtained at 20.00 ± 0.01 °C and each spectrum was acquired in 10 mmol dm^−3^ sodium phosphate buffer, pH 7.4, with an average of 16 accumulations for each spectrum acquisition.

CD spectroscopy was used to characterize the structural conformation of W-BP100 in anionic POPC:POPG (1:1) LUVs at increasing P/L ratios (Figure 1). 

The CD spectrum of W-BP100 in aqueous solution showed a random coiled conformation, as indicated by a minimum peak near 198 nm. In anionic LUVs, the CD spectra exhibited the characteristic bands of an α-helical secondary structure, namely, a positive band close to 190 nm and two negative bands of similar intensity at 208 and 222 nm, with maximum values at the P/L of 0.06. A substantial reduction in the peak intensity at P/L of 0.09 was consistent with a less ordered α-helical conformation, possibly due to the detergent-like effect of W-BP100 at this P/L ratio, leading to membrane destabilization, permeabilization, and eventually micellization [33]. The large shift in the CD spectrum at a P/L of 0.09, in the y axis direction, was attributed to an increase in the light scattering caused by vesicle aggregation. At higher P/L ratios, the peptide retained an α-helical conformation, similar to the CD spectra obtained at lower P/L (Figure 1), demonstrating that W-BP100 remains incorporated within the membrane of anionic POPC:POPG (1:1) LUVs.

The preservation of the α-helical content in anionic LUVs at P/L above 0.09, at which vesicle aggregation takes place, was further confirmed by the calculated helicity at 222 nm (Table 1). In the absence of peptide, a residual helicity was obtained, as expected by the random coil conformation exhibited in aqueous solution (Figure 1). Under vesicle aggregation conditions (P/L = 0.09), the light scattering resulting from the formation of larger particles impairs the calculation of this parameter. At higher P/L ratios, the helicity was restored to the values obtained at low P/L. Therefore, the helicity of W-BP100 was conserved independently of the P/L ratio (except at the vesicle aggregation P/L ratio).

### 3.2. Evaluation of Electroneutralization of Anionic LUVs by W-BP100

**Figure 2:**
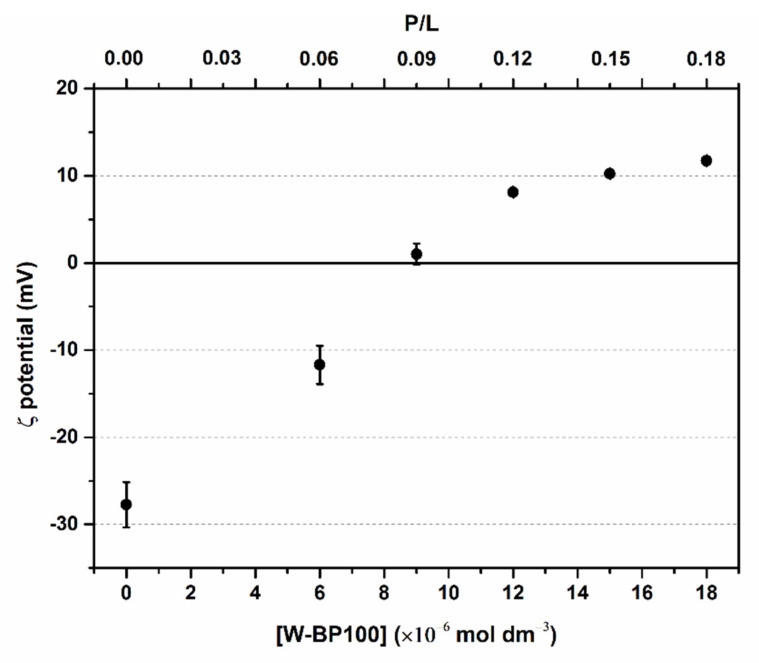
Zeta potential analysis of anionic LUVs at increasing W-BP100 concentrations. The charge of 100 µmol dm^−3^ POPC:POPG (1:1) LUVs was measured in the absence and presence of W-BP100 in HEPES buffer, pH 7.4, at 25.0 ± 0.1 °C. Data are the mean ± SD of two independent experiments.

The effect of W-BP100 on the membrane surface charge of the POPC:POPG (1:1) LUVs was assessed by zeta potential analysis to confirm whether electroneutralization of the vesicles was part of the MOA of this peptide. In the absence of W-BP100, the measured zeta potential of the anionic LUVs was around −28 mV (Figure 2), as reported for this lipid system by other authors [20,21]. A decrease in the absolute value of the zeta potential with increasing peptide concentration was observed, and a charge neutralization effect occurred (~0 mV) at a P/L of 0.09 (Figure 2), commonly (but not exclusively) associated with vesicle aggregation events [8,20,21,34,35]. At higher peptide concentrations, a shift in the zeta potential toward positive values was observed, stabilizing around +10 mV, suggesting the occurrence of hydrophobic peptide–membrane interactions upon neutralization of the negative charges of POPC:POPG (1:1) LUVs. The same effect was observed for the NK-2 peptide antibiotic in the 1,2-dipalmitoyl-*sn*-glycero-3-phospho-(1′-*rac*-glycerol) (sodium salt) (DPPG) liposomes [36] and for pepR in the POPC:POPG (4:1) and POPC:POPG (3:2) LUVs [37]. This variation in the zeta potential in the presence of W-BP100 not only supports an electrostatic driven interaction with the anionic lipid membranes, in agreement with previous work, demonstrating that W-BP100 can be inserted into the anionic LUVs, modifying the effective charge at their surface without substantially affecting vesicle size at high P/L ratios [7]. Given the well-known preference of Trp for the membrane–water interface [9,10], the observed increase in the zeta potential might indicate that W-BP100 is accommodated at the lipid membrane, with its positively charged Lys residues exposed to the external aqueous environment of liposomes.

### 3.3. Effect of W-BP100 on the Size of Anionic LUVs Overtime

**Figure 3:**
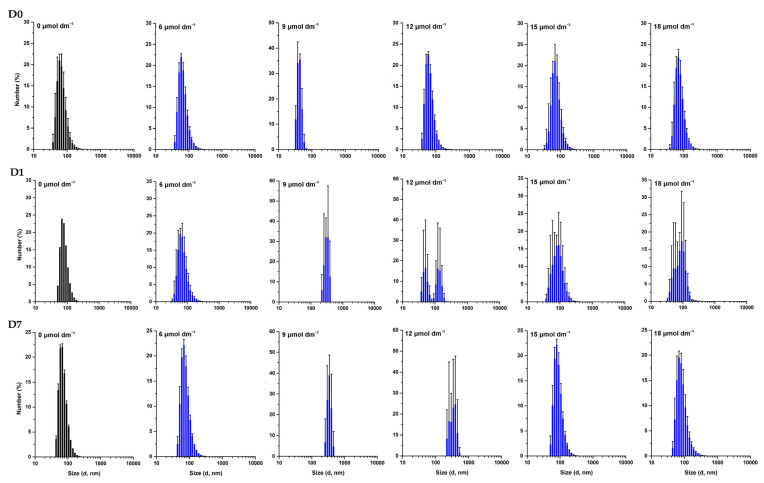
Size distribution of POPC:POPG (1:1) LUVs in the presence of W-BP100 obtained by DLS. Number-weighted size distribution of 100 µmol dm^−3^ POPC:POPG (1:1) LUVs in the absence (black) and presence (blue) of increasing W-BP100 concentrations, at days 0 (10 min upon peptide incubation), 1, and 7 upon peptide incubation, at 25.0 ± 0.1 °C. *d* stands for vesicle diameter. Data are the mean ± SD of three replicate measurements (one single experiment).

**Figure 4:**
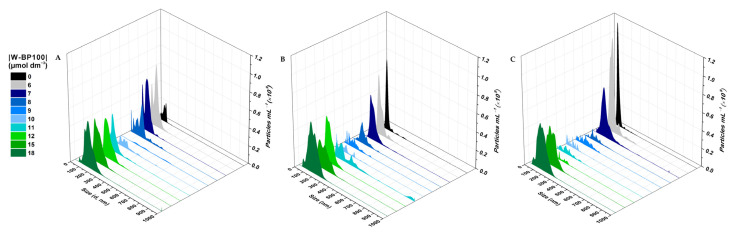
Size distribution of the POPC:POPG (1:1) LUVs in the presence of W-BP100 obtained from NTA measurements. Representative graphs of particle concentration as a function of size of 100 µmol dm^−3^ POPC:POPG (1:1) LUVs at increasing W-BP100 concentrations, 25.0 ± 0.1 °C. Size distribution was measured at (**A**) day 0 (10 min upon peptide incubation), (**B**) day 1, and (**C**) day 7 upon peptide incubation. Data represent the mean of a maximum of 10 image fields.

The effect of W-BP100 on the size of POPC:POPG (1:1) LUVs was followed over time, up to seven days of incubation, by DLS (Figure 3) and NTA (Figure 4). DLS data showed no significant alterations in the vesicle size 10 min upon peptide addition, except for a slight decrease in the mean vesicle size at 9 µmol dm^−3^ (P/L of 0.09) (Figure 3), as previously reported [7], and consistent with a detergent-like effect of the peptide, leading to the formation of smaller vesicles or micelles [4]. An increase in larger particles in solution was observed at days 1 and 7, particularly at 9 and 12 µmol dm^−3^ (P/L of 0.09 and 0.12) (Figure 3). This increase was also verified by the intensity-weighted size distribution data (Figure S1). At high P/L ratios (P/L of 0.15 and 0.18), the vesicle size distribution remained unchanged over time, similar to the size of the anionic LUVs in the absence of W-BP100 (~100 nm).

These results were further confirmed by NTA analysis, as depicted in Figure 4, demonstrating that W-BP100 does not induce significant variation in the size of anionic LUVs at higher concentrations (P/L of 0.15 and 0.18) upon 7 days of incubation. Nevertheless, we observed an increase in the size of the particles in solution and a reduction in the particle’s concentration over time for intermediate W-BP100 concentrations (P/L of 0.07–0.12). These results are attributed to vesicle aggregation as a consequence of electroneutralization of the liposome surface charge, also being consistent with a detergent-like action at such P/L ratios, possibly leading to vesicle disruption (Figure 4) [33]. The opposite was observed at increased peptide concentrations, suggesting that W-BP100 might contribute to the stabilization of POPC:POPG (1:1) LUVs at higher P/L ratios in HEPES buffer for at least one week upon peptide incubation.

**Figure 5:**
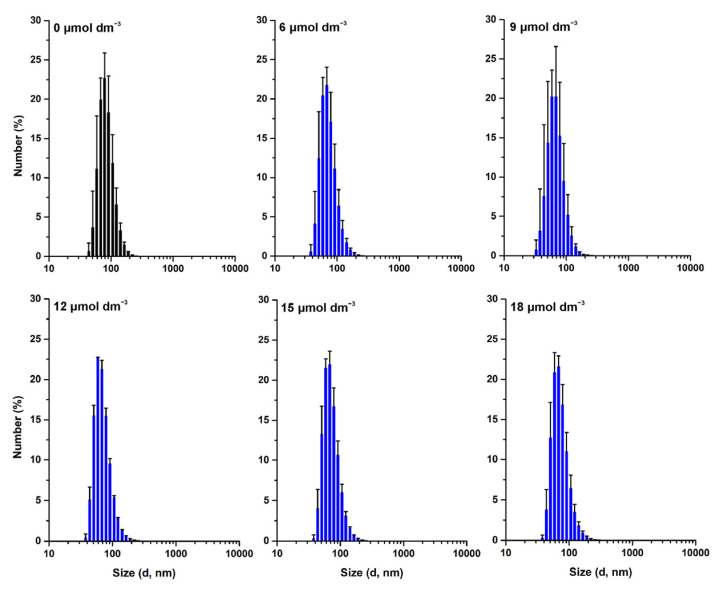
Size distribution of the POPC:POPG (3:1) LUVs in the presence of W-BP100 from the DLS measurements. Number-weighted size distribution of 100 µmol dm^−3^ POPC:POPG (3:1) LUVs in the absence and presence of increasing W-BP100 concentrations (6–18 µmol dm^−3^), upon 10 min of incubation, at 25.0 ± 0.1 °C. Data are the mean ± SD of three replicate measurements (one single experiment).

**Figure 6:**
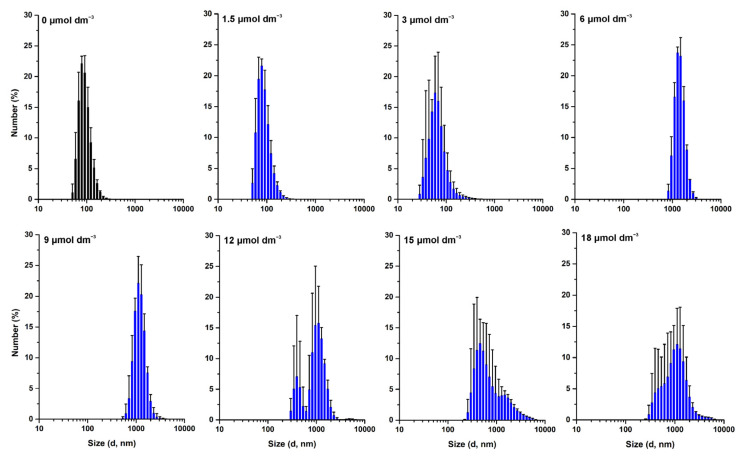
Size distribution of the POPE:POPG (1:1) LUVs in the presence of W-BP100 from the DLS measurements. Number-weighted size distribution of 100 µmol dm^−3^ POPE:POPG (1:1) LUVs in the absence and presence of increasing W-BP100 concentrations (1.5–18 µmol dm^−3^), upon 10 min of incubation, at 25.0 ± 0.1 °C. Data are the mean ± SD of three replicate measurements (one single experiment).

The effect of W-BP100 on the size of other lipid systems—POPC:POPG (3:1) and POPE:POPG (1:1)—was similarly evaluated, aiming to perceive the contribution of lipid composition, namely, the charge and curvature, on the action of this peptide. DLS data demonstrated that W-BP100 did not induce the aggregation of vesicles composed of POPC:POPG (3:1) up to a P/L ratio of 0.18 (Figure 5 and Figure S2), while the opposite was observed for POPE:POPG (1:1) LUVs (Figure 6 and Figure S3). 

These data suggest that the effect of W-BP100 on the vesicle size of POPC:POPG (1:1) LUVs is dependent on the molar ratio of the phosphatidylglycerol (PG) content (lipid charge) as well as on the presence of phosphatidylcholine (PC) (lipid topology) [38]. In the presence of zwitterionic phosphatidylethanolamine (PE) phospholipids, W-BP100 induced the irreversible aggregation of LUVs at a lower concentration (6 µmol dm^−3^; P/L of 0.06) than in vesicles composed of POPC:POPG (1:1) LUVs (Figure 6 and Figure S3). Such behavior indicates a higher affinity of WBP100 toward POPE:POPG (1:1) vs. POPC:POPG (1:1) LUVs, not solely driven by electrostatic interactions, but also by differences in the lipid topology and lipid bilayer organization.

It is known that the charge, hydrophobicity, and amphipathic nature of CAMPs are key players in their interactions with lipid membranes [4], but it is also true that lipid composition strongly influences peptide behavior and function [38,39]. The charge and amphipathic helical secondary structure of CAMPs commonly dictate their adsorption and insertion into lipid membranes, which can greatly impact their mechanical and thermotropic properties. These peptide–membrane interactions can alter lipid packing, membrane curvature, and fluidity, ultimately leading to membrane disruption and lysis [40].

Membrane-active peptides such as many CAMPs can cause deformation of membrane curvature, particularly in membranes rich in phospholipids with intrinsic negative curvature such as POPE and cardiolipin [41], but the opposite also occurs. The membrane curvature and its interfacial properties can influence peptide binding, insertion, and even secondary structure formation [41]. For instance, the negative curvature of POPE phospholipids increases the membrane fluidity by reducing lipid packing, and facilitating peptide insertion [42]. On the other hand, POPG phospholipids possess a vesicle-condensing ability, making lipid vesicles containing POPC:POPG lipids more tightly packed and rigid than the POPE:POPG mixtures. In fact, the latter are reported to increase lipid packing, promoted by the steric conformation of the large POPG glycerol headgroup, positioned on top of the small headgroup of POPE phospholipids [43]. Such physical constraints might hamper peptide insertion within the lipid bilayer of POPE:POPG (1:1) LUVs, despite allowing for electrostatic-driven adsorption to the POPG headgroups, possibly inducing surface charge neutralization and vesicle aggregation (independent of the P/L ratio).

### 3.4. Vesicle Fusion by Fluorescence Spectroscopy Using a FRET-Based Assay

The fusion activity of W-BP100 in the POPC:POPG (1:1) LUVs was assessed by steady-state fluorescence spectroscopy using a FRET-based assay [44]. Vesicle fusion was estimated by quantifying the efficiency of energy transfer or FRET efficiency (*E*) between the NBD-DOPE (donor) and Rho-DPPE (acceptor) FLL probes [32,45], incorporated into the anionic LUVs. Two experimental approaches were tested, the fluorophore mixing method and the fluorophore dilution method, as described by Bagheri et al. [46]. In the fluorophore mixing method, single-labelled LUVs containing NBD or Rho FLL probes were mixed at a 1:1 molar ratio, and as a result of FRET, a decrease in NBD and increase in the Rho fluorescence intensities were expected, translating into an increase in FRET efficiency. In the fluorophore dilution method, double-labelled LUVs containing both NBD and Rho probes were mixed with unlabelled vesicles at a 1:2 molar ratio, conducive to an increase in NBD and a decrease in the Rho fluorescence intensities, leading to FRET reduction.

**Figure 7:**
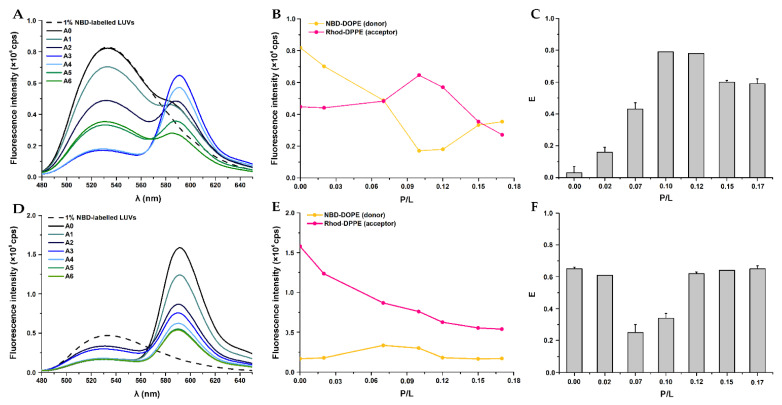
Evaluation of the fusion activity of W-BP100 in anionic LUVs by FRET using steady-state fluorescence spectroscopy. Two methodologies were tested: (**A**–**C**) the fluorophore mixing method and (**D**–**F**) the fluorophore dilution method. (**A**,**D**) Representative emission fluorescence spectra of NBD-DOPE and Rho-DPPE labelled LUVs in the absence (A0) and presence of increasing W-BP100 concentrations (A1–A6). Fluorescence spectra of single-labelled NBD-LUVs (donor) are represented by a dashed line. (**B**,**E**) Representative maximum fluorescence emission of NBD-DOPE (λ_em_ = 530 nm) and Rho-DPPE (λ_em_ = 590 nm) as a function of the P/L ratio. (**C**,**F**) FRET efficiency (*E*) between NBD-DOPE (donor) and Rho-DPPE (acceptor) as a function of the P/L ratio was calculated by Equation (2). Data are the mean ± SD of two independent experiments.

The effect of W-BP100 on the fusion of POPC:POPG (1:1) LUVs is reported in Figure 7.

In the absence of W-BP100, spontaneous vesicle fusion events were negligible, as a small variation in NBD fluorescence intensity was detected when compared to the fluorescence of NBD-labelled vesicles, used as controls to calculate E, using Equation (2) (Figure 7A,D). The small differences in the fluorescence intensity of single labelled NBD-LUVs compared to double labelled NBD-/Rho-LUVs, noticed in the fluorophore dilution method, can be explained by the close proximity between the two probes, leading to a maximum FRET signal, and consequently, a slight reduction in the NBD fluorescence intensity compared with the NBD-labelled vesicles (control) [45]. In the fluorophore mixing method, decreased NBD and increased Rho fluorescence intensities were observed with increasing W-BP100 concentration (Figure 7A–C), consistent with an increase in FRET efficiency in a dose-dependent manner up to a P/L ratio of 0.12 (Figure 7C). These results suggest that W-BP100 induced moderate fusion of the anionic lipid vesicles at lower concentrations before reaching the membrane saturation conditions (P/L of ~0.06) [7]. At the P/L of 0.10–0.12, at which vesicle aggregation takes place (Figure 1 and Figure 3), the highest values of FRET efficiency were obtained, indicating that aggregation and fusion are closely associated events. At higher P/L ratios, FRET was slightly reduced but not abolished, suggesting that the NBD and Rho probes were positioned at close distance within the lipid vesicles. Interestingly, these lipid vesicles resemble the size of anionic LUVs in the absence of peptide, showing an average size of ~100 nm (Figure 3). In the fluorophore dilution method (Figure 7D–F), vesicle fusion was mainly observed in the P/L range 0.07–0.10, with a ~2-fold reduction in the FRET efficiency (Figure 7F), again occurring predominantly at vesicle aggregation conditions. The recovery of FRET at higher P/L ratios suggests vesicle and/or lipid rearrangement upon LUV aggregation, which favored the proximity of the NBD and Rho probes, rescuing the FRET efficiency to values obtained in the absence of peptide.

FRET assays showed that W-BP100 induced low to moderate fusion of anionic POPC:POPG (1:1) LUVs at low P/L ratios, largely increasing at intermediate P/L, as a result of vesicle aggregation (Figure 7C,F). High and similar FRET efficiencies were obtained in both methodologies at high P/L ratios, suggesting that upon vesicle aggregation, the NBD and Rho probes were repositioned at close proximity, allowing FRET to occur.

Bagheri et al. investigated the aggregation and fusion activity of peptides HHC-10 and ^4Har^HHC-10, rich in Arg and Trp residues, in membrane models containing phospholipids with different charge and curvature properties [46]. They observed that peptides induced a similar pattern of vesicle aggregation and disaggregation, with no vesicle fusion in the POPC:POPG (1:3) LUVs, while in POPE:POPG (3:1), only aggregation was observed. The authors attributed the aggregation/disaggregation behavior to the re-establishment of the surface charge of anionic vesicles resultant from peptide translocation, thus evading the aggregation effect [46]. The same effect was early observed for the CPP penetratin in DOPG LUVs by Persson et al. [47], and more recently for the CPP EB1 in anionic LUVs by Svirina et al. [48]. Analyzing the penetratin example, this peptide caused the aggregation of anionic vesicles, but at high P/L ratios, the vesicles showed a size similar to that of liposomes not exposed to the peptide. This was attributed to spontaneous vesicle dissociation and not induced by vesicle fusion. Among the possible hypotheses to explain such an effect, the most plausible was peptide translocation, leading to peptide depletion from the outer lipid leaflet, reversing the membrane surface charge. This effect successively occurred with an increasing concentration of penetratin, promoting new vesicle aggregation and dissociation up to a certain P/L ratio (where dissociation and thus possible translocation do not occur anymore) [47]. We observed the same aggregation and possible disaggregation of the POPC:POPG (1:1) LUVs by DLS, but we verified a stabilization of the vesicle size with an increasing peptide concentration. Moreover, we observed vesicle fusion at the aggregation P/L ratio, which possibly impacts the effect of W-BP100 on the size and reorganization of lipid vesicles.

Although vesicle aggregation and fusion are not always correlated, there is a number of CAMPs and CPPs whose MOA involves these two processes. Domingues et al. demonstrated that the rBPI21 fusion peptide destabilizes POPG membranes by a charge- and fusion-dependent manner [49]. The peptide induces vesicle aggregation and membrane fusion or hemi-fusion, leading to membrane permeabilization and content leakage in the POPG-containing membranes. This effect was even more pronounced in LUVs containing liposaccharide (LPS), but PG phospholipids are required for the occurrence of membrane fusion [49]. The same preference for anionic lipids in membrane fusion events was observed by Stauffer et al. with the dengue virus fusion peptide DEN Fpep [50]. The authors showed that fusion was more efficient in the presence of anionic lipids and favored at high P/L ratios by peptide oligomerization and clustering within the lipid vesicles [50].

### 3.5. Confocal Microscopy Analysis of Aggregation and Fusion of Anionic GUVs

Confocal microscopy studies were performed to characterize the action of W-BP100 using fluorescently-labelled GUVs. GUVs are an attractive system to study peptide–membrane interactions given their size (close to the size of bacterial cells), allowing for the detailed visualization of the structure and dynamics of lipid membranes by fluorescence microscopy [27,51,52].

**Figure 8:**
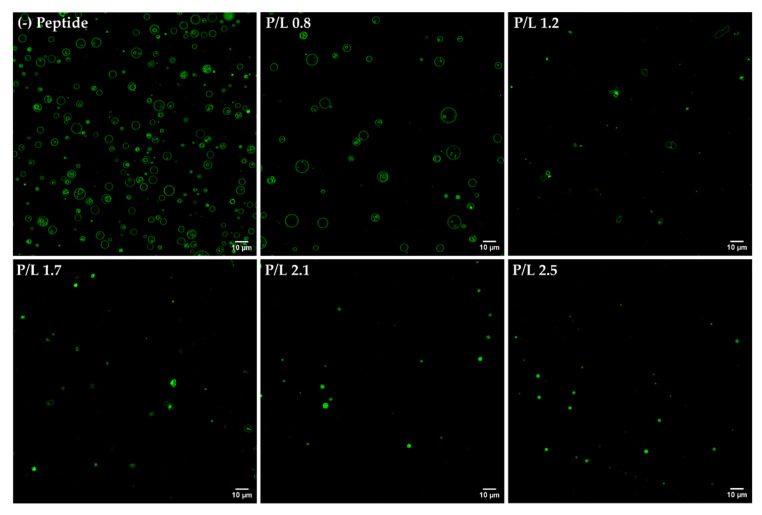
Morphology of the POPC:POPG (1:1) GUVs in the presence of W-BP100 obtained by confocal microscopy. Representative images of 2% NBD-labelled GUVs in the absence (−) and presence of peptide (P/L of 0.8, 1.2, 1.7, 2.1, and 2.5) upon 10 min of incubation. GUVs were incubated with HEPES buffer (solvent of the peptide) to exclude any contribution to GUV aggregation (Figure S5). Videos of W-BP100 action on GUVs are provided in the Supplementary Materials (Videos S1–S3). Magnification: 630×. Scale bar: 10 µm.

**Figure 9:**
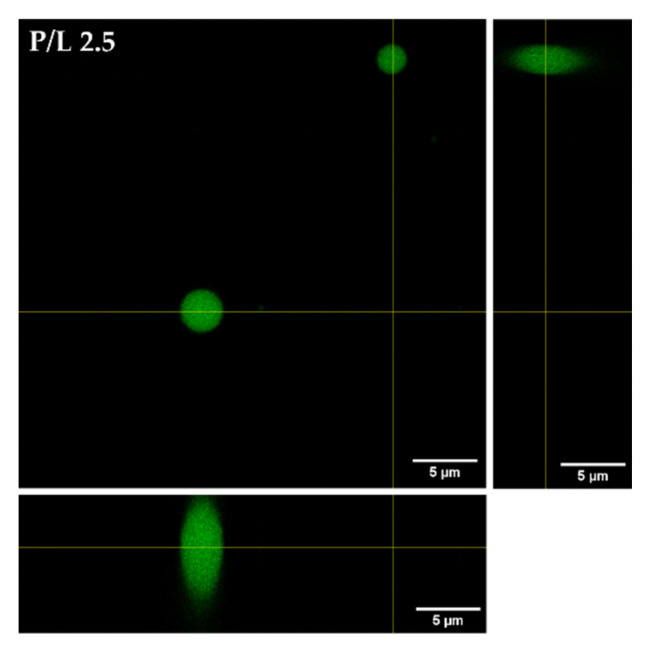
Formation of new lipid aggregates at higher W-BP100 concentration. Representative orthogonal view of lipid agglomerates formed at a 2.5 P/L ratio. Magnification: 630×, zoom 0.5×. Scale bar: 5 µm.

**Figure 10:**
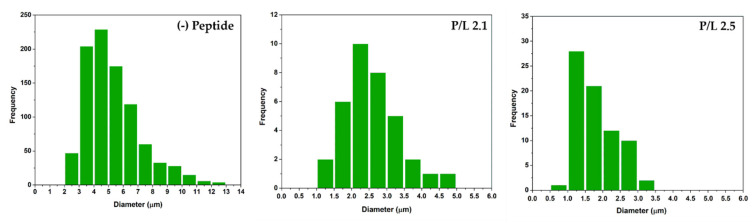
Size diameter distribution of the POPC:POPG (1:1) GUVs and the new lipid aggregates formed at high P/L ratios. The size diameter of the GUVs in the absence (−) and presence of peptide (P/L ratio of 2.1 and 2.5) was measured in ImageJ software using 4−5 images per condition, and a number of vesicles per image of 134–270 (absence of peptide; 920 total), 3–16 (2.1 P/L; 35 total), and 10–20 (2.5 P/L; 74 total).

We first evaluated the morphological transformations induced by W-BP100 binding to POPC:POPG (1:1) GUVs, labelled with 2% NBD-DPPE (Figure 8) to enable fluorescence visualization of the membrane. At a low P/L ratio (P/L = 0.8), no significant alterations in the vesicle or membrane structure were observed, with preservation of the typical unilamellar and round-shaped structure of GUVs (Figure 8, Video S1). With increasing W-BP100 concentration, there was a reduction in the quantity of intact GUVs in the P/L ratio range of 1.2–1.7. The remaining GUVs lost their spherical surface (Figure S4B,C), exhibiting a distorted shape (in some cases showing elongated tubular structures). This change may arise from the incorporation of the peptide within the membrane, leading to an increase in the membrane fluidity. Formation of NBD-rich lipid domains were also observed, pointing to lipid segregation at the membrane. W-BP100 seems to disrupt the membrane of GUVs, leading to their destruction (Figure S4A), and ultimately inducing the aggregation of small vesicle-like structures, resembling a detergent-like MOA (Video S2). At high P/L ratios (P/L = 2.1 and 2.5), W-BP100 induced the formation of new lipid structures with an amorphous, dense, and oval shape, as illustrated in Figure 9. These new lipid aggregates were smaller in size (average size diameter: 1−3 μm at the P/L of 2.5) compared to the initial GUV size (average size diameter: 3−6 μm), as depicted in Figure 10. This behavior may arise from the aggregation of membrane fragments and small lipid vesicles still remaining in solution triggered by W-BP100 at high P/L ratios (Video S3). Importantly, the W-BP100 action was similar in both LUVs and GUVs, although at distinct P/L ratios. The differences found might be caused by the higher surface area of GUVs, requiring higher amounts of peptide to obtain a similar effect on the vesicle size and morphology.

The behavior of W-BP100 in POPC:POPG (1:1) GUVs resembled the effect of the parent CAMP, BP100, on the anionic GUVs up to the P/L ratio of vesicle aggregation, disrupting anionic membranes [21]. Carretero et al. showed that BP100 and its cyclic derivative mainly promoted vesicle burst, while a BP100 derivative holding a 16-carbon acyl chain induced extensive vesicle aggregation, followed by GUV disruption and burst [20]. 

The formation of tubular lipid-like structures and vesicle budding are typical of several membrane-active peptides such as the recently discovered LBF14 14-mer peptide [53]. This peptide was found to induce the drastic morphological disruption of membranes, independently of lipid composition. Its membrane activity was attributed to membrane insertion at the membrane–water interface, inducing local curvatures and membrane remodeling through vesicle bursting and the formation of interconnected tubules and small membrane vesicles [53].

Vesicle fusion studies using a mixture of GUVs labelled with 2% NBD-DPPE or 0.1% Rho-DPPE (1:1 molar ratio) were performed to evaluate the ability of W-BP100 to induce the fusion of anionic POPC:POPG (1:1) GUVs. 

**Figure 11:**
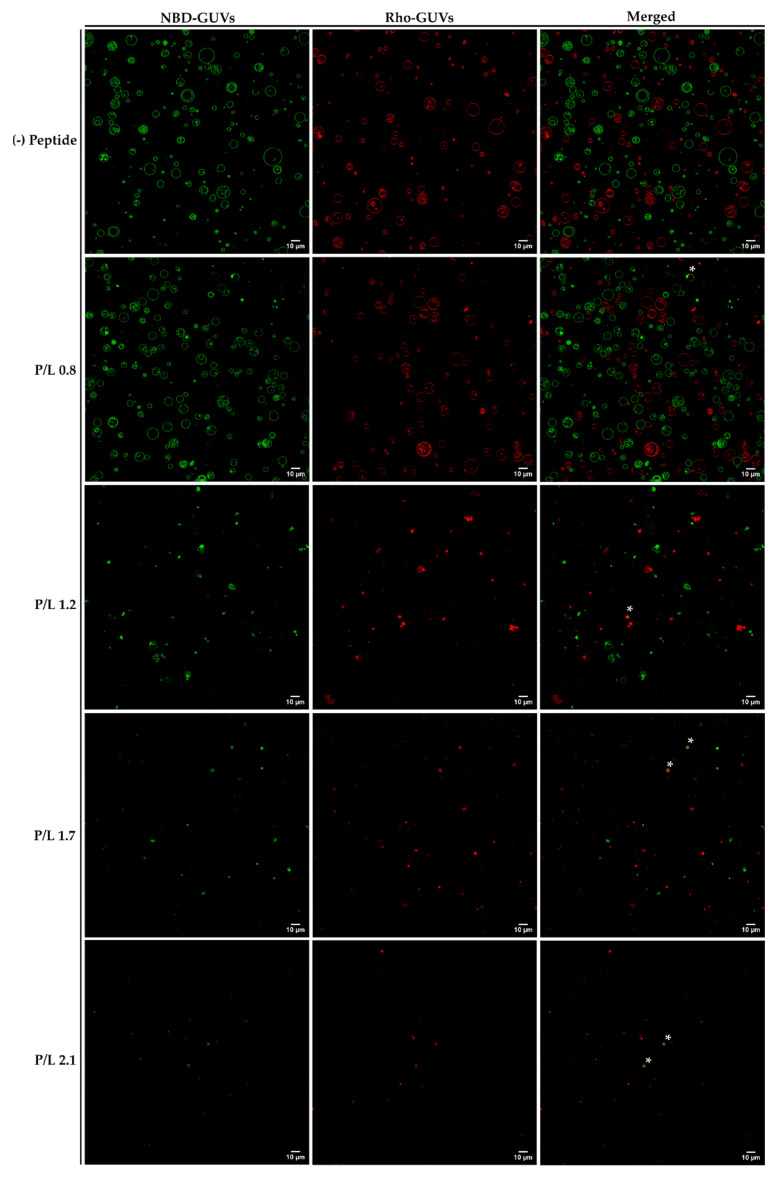
Fusion of the POPC:POPG (1:1) GUVs in the presence of W-BP100 by confocal microscopy. Representative images of a mixture of 2% NBD- and 0.1% Rho-labelled GUVs (1:1 molar ratio) in the absence (−) and presence of peptide (P/L of 0.8, 1.2, 1.7 and 2.1). Fusion events are highlighted with asterisks (*). Magnification: 630×. Scale bar: 10 µm.

At a low P/L ratio (P/L = 0.8), the fusion of GUVs by the action of W-BP100 was rarely observed (highlighted with asterisks in Figure 11). At this P/L ratio, it was possible to observe lipid protrusions from intact GUVs as well as the budding and mixing of vesicles, although essentially between GUVs labelled with the same FLL.

When increasing the peptide concentration (P/L = 1.2 and 1.7), the amount of vesicle aggregates labelled with NBD or Rho FLLs increased in a concentration-dependent manner (Figure 11). However, the appearance of lipid vesicles or aggregates with overlapping fluorescence of NBD and Rho was still scarce (highlighted with asterisks in Figure 11). In addition, lipid domains enriched in FLLs were again observed (Figure 11, P/L = 1.2), confirming that W-BP100 induces alterations in lipid dynamics and fluidity.

Finally, at high P/L ratios (P/L = 2.1 and 2.5), the morphology of the newly formed lipid aggregates was similar to the one previously obtained with NBD FLLs (Figure 8). Instead, two populations with comparable morphology and size (either labelled with NBD or Rho FLLs) were observed (Figure 11). Importantly, at high P/L (P/L = 2.1), fluorescence quenching of NBD and Rho FLLs was observed, suggesting a quenching effect attributed to either Trp [17] or to high peptide concentrations used (~100 μmol dm^−3^) [18,44]. This artifact impaired image acquisition at a P/L of 2.5.

Remodeling of lipid vesicles by the action of membrane-active peptides or proteins has not been a recent topic in the scientific literature. The α-synuclein protein is known to accumulate as amyloid aggregates in neuronal tissue of Parkinson’s disease patients but is also able to interact with membranes under physiological and pathological scenarios. Mizuno et al. reported the ability of α-synuclein to remodel anionic lipid vesicles at certain P/L ratios, transforming spherical vesicles into cylindrical micelles [54]. At even higher P/L ratios, the protein is able to generate discoid particles that are double in size. Such dramatic alterations in the vesicle morphology induced by α-synuclein could support the morphological transformations originated by W-BP100 in the POPC:POPG (1:1) GUVs. The amorphous lipid aggregates formed at high P/L ratios might concentrate peptide-lipid clusters, and thus could work as a possible peptide delivery agent.

In summary, confocal data support a detergent-like action of W-BP100 at critical P/L ratios, followed by the aggregation of small lipid vesicles and lipid remodeling, ultimately leading to the formation of new and stable lipid aggregates [4].

## 4. Conclusions

The aggregation and fusion properties of W-BP100, a new and potent CAMP, were explored in anionic bacterial membrane models using a multidisciplinary biophysical approach. CD experiments evidenced an α-helical conformation of W-BP100 in anionic bilayers below and above the critical P/L ratio at which vesicle aggregation occurs. Zeta potential measurements showed that W-BP100 induced the electroneutralization of the surface charge of anionic membrane bilayers. W-BP100 was found to promote the aggregation and fusion of negatively charged liposomes, dependent on the P/L ratio. DLS and NTA data showed that W-BP100 induces the aggregation of anionic lipid vesicles at a critical P/L ratio, above which the size of the obtained particles drastically reduces to the sizes of the initial lipid vesicles. Fluorescence experiments (steady-state spectroscopy and confocal microscopy) corroborated the vesicle aggregation and fusion in the presence of W-BP100, mainly at membrane saturation/aggregation conditions.

Overall, W-BP100 seems to promote a reorganization of the anionic lipid bilayer at a critical P/L ratio, possibly by acting through a detergent-like mechanism, above which it is able to reshape lipid vesicles into new lipid aggregates. These new lipid structures may contain embedded peptide molecules in their composition, as reported for other CAMPs. Therefore, the location and conformation of W-BP100 within these new lipid aggregates should be further explored. The contribution of the aggregation and fusion activities to the antibacterial action of W-BP100 also requires additional studies to perceive how or if the obtained data in the model membranes translate into a similar behavior at the bacterial cell level.

Combining the results obtained in this work with the antibacterial activity data previously reported [7], it is possible to infer that W-BP100 is a promising CAMP to fight bacterial infections caused by both Gram-negative or Gram-positive bacteria. W-BP100 was proven to have a potent broad-spectrum antibacterial activity, being substantially more potent than its parent peptide BP100 against the Gram-positive strains.

## Figures and Tables

**Table 1:** Helicity ($f_{H}$) of W-BP100 in POPC:POPG (1:1) LUVs at increasing P/L ratios. Measurements were made at 222 nm in 10 mmol dm^−3^ sodium phosphate buffer, pH 7.4, 20 °C, and $f_{H}$ was calculated using Equation (2).

P/L	0.00	0.03	0.06	0.09	0.12	0.15
$f_{H}$ (%)	12	71	81	--- *	67	74

* Vesicle aggregation impairs the calculation of the helicity.

## Data Availability

Not applicable.

## Supplementary Materials

The following supporting information can be downloaded at: https://www.mdpi.com/article/10.3390/membranes13020138/s1, Figure S1: Intensity-weighted size distribution of POPC:POPG (1:1) LUVs in the presence of W-BP100 obtained by DLS; Figure S2: Intensity-weighted size distribution of POPC:POPG (3:1) LUVs in the presence of W-BP100 obtained by DLS; Figure S3: Intensity-weighted size distribution of the POPE:POPG (1:1) LUVs in the presence of W-BP100 obtained by DLS; Figure S4: Confocal microscopy imaging of the POPC:POPG (1:1) GUVs incubated with W-BP100 (P/L = 1.2); Figure S5: Confocal microscopy imaging of the POPC:POPG (1:1) GUVs incubated with HEPES buffer; Video S1: Confocal microscopy imaging of the POPC:POPG (1:1) GUVs incubated with W-BP100 (P/L = 0.8); Video S2: Confocal microscopy imaging of the POPC:POPG (1:1) GUVs incubated with W-BP100 (P/L = 1.7); Video S3: Confocal microscopy imaging of the POPC:POPG (1:1) GUVs incubated with W-BP100 (P/L = 2.5).
