# Supplementary material for: The Unusual Aggregation and Fusion Activity of the Antimicrobial Peptide W-BP100 in Anionic Vesicles
Source: Membranes (Basel). 2023 Jan 21;13(2):138. doi: 10.3390/membranes13020138 (PMC9966869; doi:10.3390/membranes13020138)
Supplement: Supplementary file 1 [file membranes-13-00138-s001.zip › membranes-2116149-supplementary/membranes-2116149-supplementary.pdf]

SUPPLEMENTARY MATERIAL

The unusual aggregation and fusion activity of the antimicrobial peptide W-BP100 in anionic vesicles

Ana Rita Ferreira [1], Mariana Ferreira [1,*], Claúdia Nunes [2], Salette Reis [2], Cátia Teixeira [1,†], Paula Gomes [1] and Paula Gameiro [1]

1 LAQV/REQUIMTE (Laboratório Associado para a Química Verde—Rede de Química e Tecnologia), Departamento de Química e Bioquímica, Faculdade de Ciências, Universidade do Porto, Rua do Campo Alegre, s/n, 4169-007 Porto, Portugal; ritaferreira@fc.up.pt (A.R.F.); pgomes@fc.up.pt (P.G.); agsantos@fc.up.pt (P.G.).

2 LAQV/REQUIMTE, Laboratório de Química Aplicada, Faculdade de Farmácia da Universidade do Porto, Portugal, Rua de Jorge Viterbo Ferreira, 228, 4050-313 Porto, Portugal; cdnunes@ff.up.pt (C.N.); shreis@ff.up.pt (S.R.).

† Current affiliation: Gyros Protein Technologies, Tucson, AZ 85714, United States of America. ca.teixeira@gmail.com (C.T.).

* Correspondence: mariana.ferreira@fc.up.pt (M.F.).

Contents of Supplementary material

Supplementary Figure S1. Intensity-weighed size distribution of POPC:POPG (1:1) LUVs in the presence of W-BP100 obtained by DLS.

Supplementary Figure S2. Intensity-weighed size distribution of POPC:POPG (3:1) LUVs in the presence of W-BP100 obtained by DLS.

Supplementary Figure S3. Intensity-weighed size distribution of POPE:POPG (1:1) LUVs in the presence of W-BP100 obtained by DLS.

Supplementary Figure S4. Confocal microscopy imaging of POPC:POPG (1:1) GUVs incubated with W-BP100 (P/L = 1.2).

Supplementary Figure S5. Confocal microscopy imaging of POPC:POPG (1:1) GUVs incubated with HEPES buffer.

Supplementary Video S1. Confocal microscopy imaging of POPC:POPG (1:1) GUVs incubated with W-BP100 (P/L = 0.8).

Supplementary Video S2. Confocal microscopy imaging of POPC:POPG (1:1) GUVs incubated with W-BP100 (P/L = 1.7).

Supplementary Video S3. Confocal microscopy imaging of POPC:POPG (1:1) GUVs incubated with W-BP100 (P/L = 2.5).

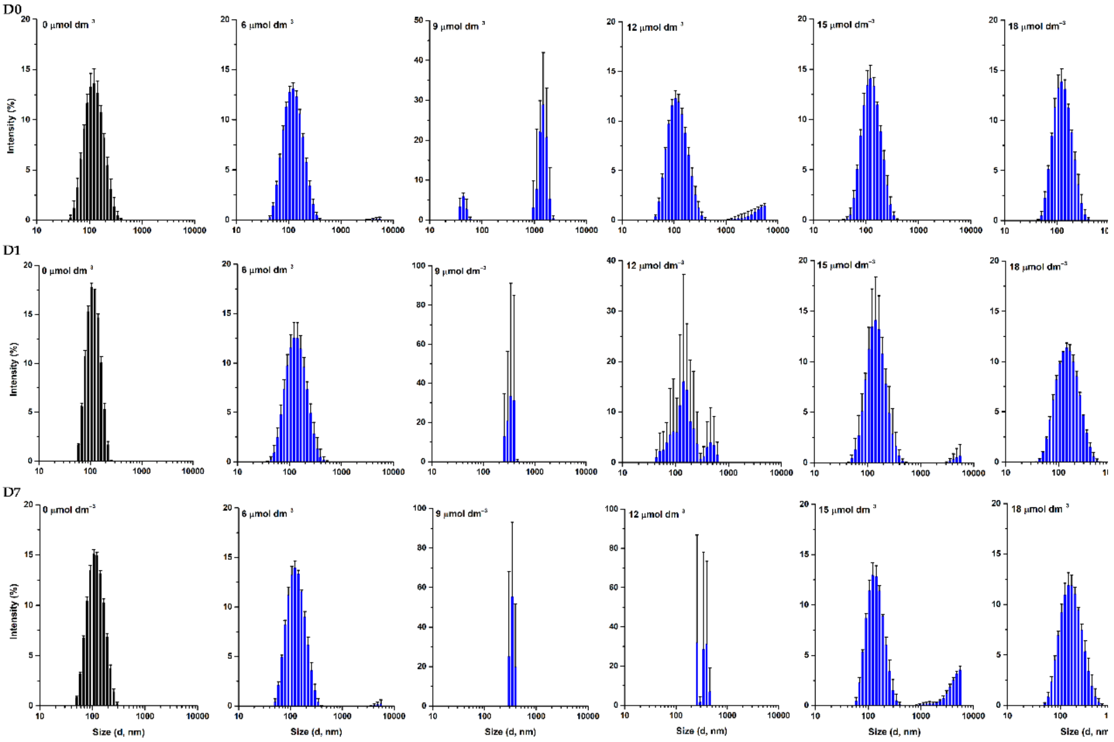

Figure S1. Intensity-weighed size distribution of POPC:POPG (1:1) LUVs in the presence of W-BP100 obtained by DLS. Intensity-weighed size distribution of 100 µmol dm^{-3} POPC:POPG (1:1) LUVs in the absence (black) and presence (blue) of increasing W-BP100 concentrations, at day 0 (10 min upon peptide incubation), 1 and 7 upon peptide incubation, at 25.0 ± 0.1 °C. *d* stands for vesicle diameter. Data are the mean ± SD of three replicate measurements (one single experiment).

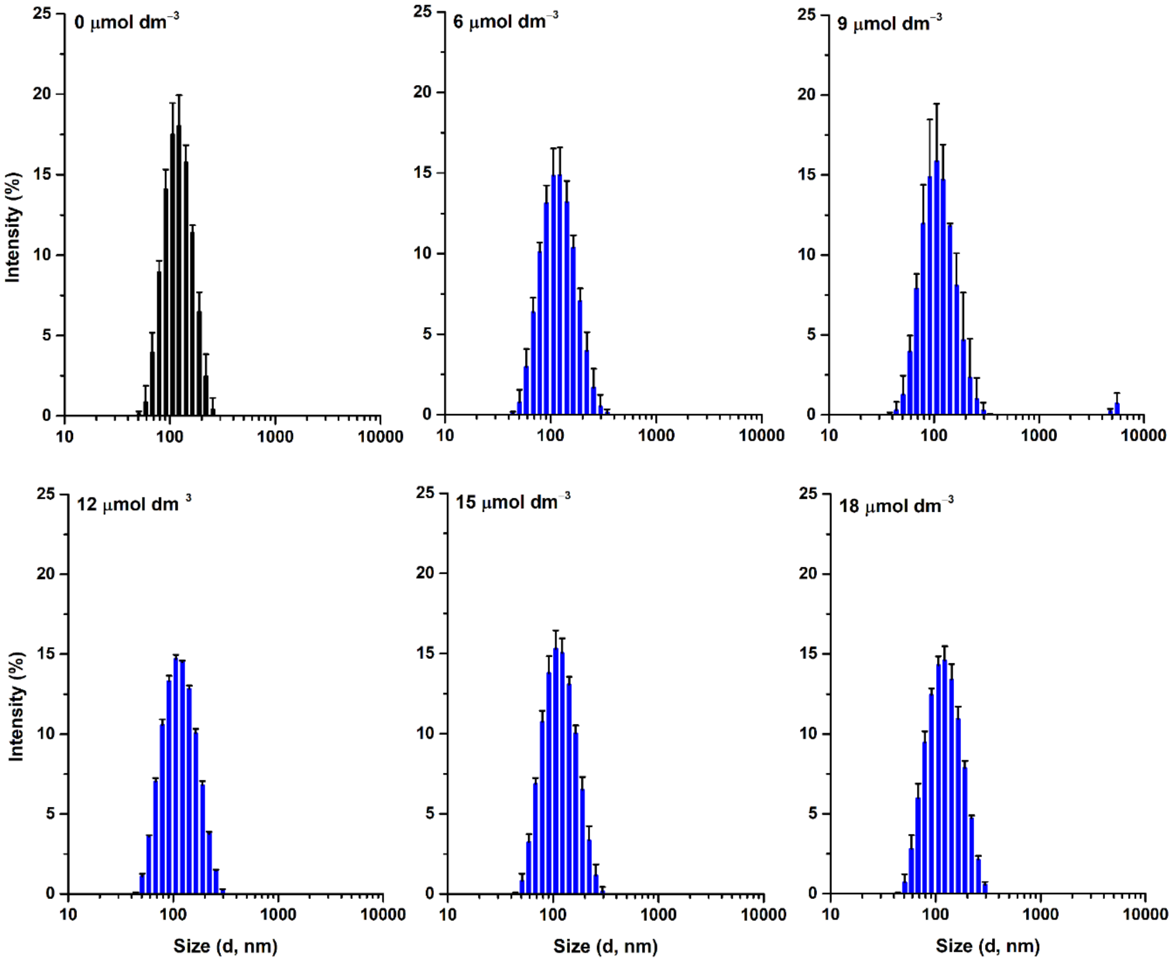

Figure S2. Intensity-weighed size distribution of POPC:POPG (3:1) LUVs in the presence of W-BP100 obtained by DLS. Intensity-weighed size distribution of 100 μmol dm^{-3} POPC:POPG (3:1) LUVs in the absence (black) and presence (blue) of increasing W-BP100 concentrations, at 25.0 ± 0.1 °C. *d* stands for vesicle diameter. Data are the mean ± SD of three replicate measurements (one single experiment).

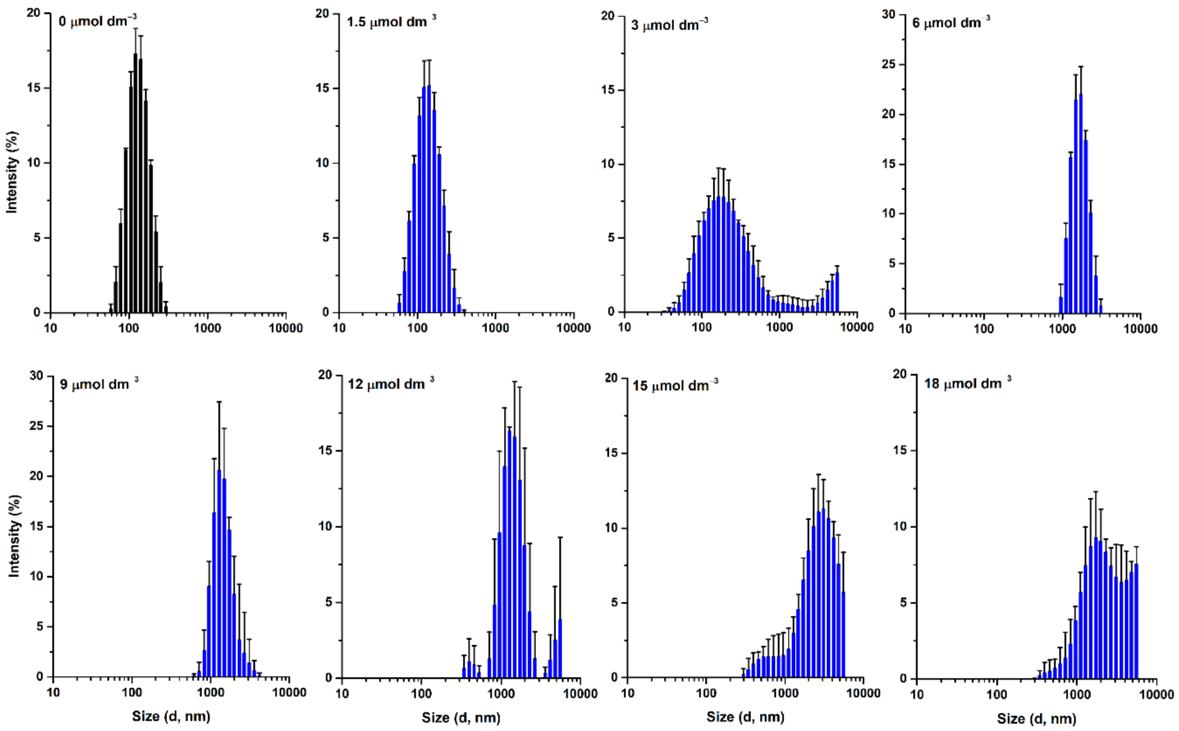

Figure S3. Intensity-weighed size distribution of POPE:POPG (1:1) LUVs in the presence of W-BP100 obtained by DLS. Intensity-weighed size distribution of 100 μmol dm^{-3} POPE:POPG (1:1) LUVs in the absence (black) and presence (blue) of increasing W-BP100 concentrations, at 25.0 ± 0.1 °C. *d* stands for vesicle diameter. Data are the mean ± SD of three replicate measurements (one single experiment).

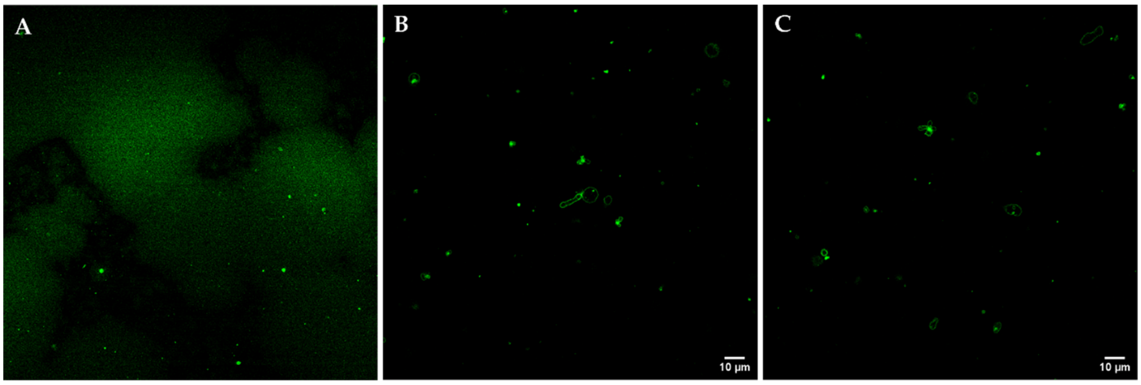

Figure S4. Confocal microscopy imaging of POPC:POPG (1:1) GUVs incubated with W-BP100 (P/L = 1.2). 2% NBD-labeled GUVs (~ 48 μmol dm^{-3}) were incubated with W-BP100 for 10 min and images were acquired under a 63×-oil objective, over 10 min, with 10 s interval between frames. (A) Adhesion of NBD FLLs to the bottom of the petri dish due to GUV destruction. (B) Formation of elongated vesicles with tubular-like morphology. (C) Formation of large aggregates constituted by small lipid-like vesicles. Magnification: 630×. Scale bar: 10 μm.

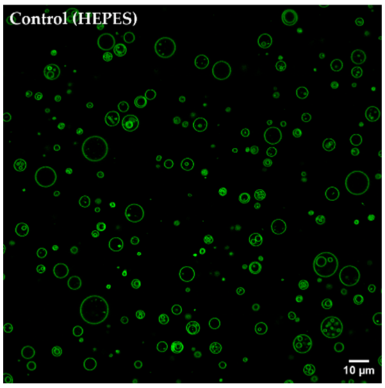

Figure S5. Confocal microscopy imaging of POPC:POPG (1:1) GUVs incubated with HEPES buffer. An equal volume of HEPES buffer, the solvent of the peptide stock solution, was added to the 40 µL drop of GUVs used for image analysis (control). The volume added (11 µL) corresponded to the total volume of peptide added to obtain the final P/L ratio tested (P/L = 2.5). Magnification: 630×. Scale bar: 10 µm.

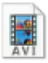

Video S1.avi

Video S1. Confocal microscopy imaging of POPC:POPG (1:1) GUVs incubated with W-BP100 (P/L = 0.8). 2% NBD-labeled GUVs (~ 48 µmol dm^{-3}) were incubated with W-BP100 for 10 min and images were acquired under a 63×-oil objective, over 10 min, with 10 s interval between frames. Magnification: 630×. Scale bar: 10 µm.

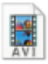

Video S2.avi

Video S2: Confocal microscopy imaging of POPC:POPG (1:1) GUVs incubated with W-BP100 (P/L = 1.7). 2% NBD-labeled GUVs (~ 48 µmol dm^{-3}) were incubated with W-BP100 for 10 min and images were acquired under a 63×-oil objective, over 10 min, with 10 s interval between frames. Magnification: 630×. Scale bar: 10 µm.

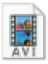

Video S3.avi

Video S3: Confocal microscopy imaging of POPC:POPG (1:1) GUVs incubated with W-BP100 (P/L = 2.5). 2% NBD-labeled GUVs (~ 48 µmol dm^{-3}) were incubated with W-BP100 for 10 min and images were acquired under a 63×-oil objective, over 10 min, with 10 s interval between frames. Magnification: 630×. Scale bar: 10 µm.
